# Oxidative Stress in Retinal Diseases

**DOI:** 10.1155/2017/4076518

**Published:** 2017-03-15

**Authors:** Yuhei Nishimura, Hideaki Hara, Mineo Kondo, Samin Hong, Takeshi Matsugi

**Affiliations:** ^1^Department of Molecular and Cellular Pharmacology, Pharmacogenomics and Pharmacoinformatics, Mie University Graduate School of Medicine, Mie 514-8507, Japan; ^2^Molecular Pharmacology, Department of Biofunctional Evaluation, Gifu Pharmaceutical University, Gifu 501-1196, Japan; ^3^Department of Ophthalmology, Mie University Graduate School of Medicine, Tsu, Mie 514-8507, Japan; ^4^Department of Ophthalmology, Yonsei University College of Medicine, Seoul 03722, Republic of Korea; ^5^Non-Clinical Research, Santen Pharmaceutical Co. Ltd., Osaka 530-8552, Japan

The retina is exposed to chronic oxidative stress (OS) through several mechanisms, including constant exposure to light and reactive oxygen species, which are generated by visual signal transduction pathways triggered by high oxygen consumption, oxidization of polyunsaturated fatty acids, and phagocytosis of photoreceptor cells. In the healthy state, all cell types in the retina are able to maintain homeostasis under conditions of OS. However, when the balance between pro- and antioxidative signaling is compromised; excessive OS induces dysregulation of functional networks and deleterious changes that result in visual impairment. Age-related macular degeneration (AMD), diabetic retinopathy (DR), and glaucoma are leading causes of visual impairment. Owing to a combination of lifestyle changes, such as increased consumption of a high-fat diet and decreased physical activity, and extended life expectancy, an increasing number of people are at risk for retinal diseases, and the resulting economic burden imposed on health care systems is increasing accordingly. This special issue focuses on the role of OS in retinal diseases.

A. Kimura et al. review the contribution of OS to glaucoma and optic neuritis. Glaucoma is characterized by progressive degeneration of retinal ganglion cells (RGC) and their axons, which form the optic nerve. Optic neuritis is a demyelinating inflammation of the optic nerve that is usually associated with multiple sclerosis. In their review, the authors focus especially on the role of apoptosis signal-regulating kinase 1 (ASK1). They previously demonstrated that ASK1 deficiency significantly reduces OS in various animal models of glaucoma and optic neuritis. Moreover, the expression of toll-like receptor 4 (TLR4) is increased in the glaucoma model, which, in turn, activates ASK1. Interestingly, candesartan, an angiotensin II receptor antagonist, suppresses the increase in TLR4 expression in the glaucoma model. The renin-angiotensin system (RAS) is reportedly involved in OS-induced RGC death. These findings suggest that suppression of RAS-TLR4-ASK1 signaling may be a promising approach to reduce OS in glaucoma and optic neuritis.

T. Matsuura et al. focus on the role of OS in AMD, which is a leading cause of blindness in developed countries. AMD is classified as wet or dry: the wet form is characterized by choroidal neovascularization (CNV) and the dry form is characterized by atrophy of retinal pigment epithelium (RPE). Multiple risk factors, including obesity, hypertension, smoking, and light exposure, have been reported to contribute to the pathogenesis of AMD by increasing OS. Therefore, it is important to identify biomarkers that reflect the level of OS and the state of AMD. Matsuura et al. examined serum levels of malondialdehyde (MDA), a well-known marker of OS, in patients with AMD and in healthy subjects. They found not only that MDA levels were significantly higher in patients with wet AMD compared to healthy subjects but also that the MDA levels correlated significantly with the state of CNV. The authors also demonstrated that nutritional supplementation with anti-OS agents tended to reduce MDA levels in patients with wet AMD. These findings suggest that MDA might be a valuable biomarker of the OS and the CNV status in wet AMD.

K. Ohashi et al. explore OS in the rat retina caused by the polyamine spermidine. Polyamines are metabolites of ornithine, and previous studies have suggested that ornithine accumulation is involved in the pathogenesis of dry AMD. In vitro studies found that high levels of spermidine induce RPE cell death; however, the underlying mechanisms are incompletely understood. K. Ohashi and colleagues examined the effects of intravitreal spermidine injection in the rat. They found that spermidine increased the permeability of the blood-retinal barrier and induced the vacuolation, atrophy, and death of RPE cells. However, these effects were profoundly inhibited by coinjection of N-acetylcysteine, an antioxidant, or of aldehyde dehydrogenase with spermidine. These results suggest that intravitreal injection of spermidine causes dry AMD by increasing OS.

C. Li et al. review the contribution of OS to DR, which is a major and potentially life-threatening complication of diabetes and is the main cause of blindness among working-age adults. Four classical pathways are involved in the pathophysiology of DR: increased polyol pathway flux, activation of the protein kinase C pathway, accumulation of advanced glycation end products, and activation of the hexosamine pathway. C. Li et al. demonstrate that hyperglycemia also affects epigenetic regulatory pathways, including promoter methylation, histone acetylation, and microRNA expression. Dysregulation of epigenetic pathways by hyperglycemia can affect the expression of genes related to OS in the retina and could thus play a critical role in the development of DR.

S. Balaiya et al. also review the involvement of OS-induced epigenetic changes in retinal diseases, focusing on the sirtuins, which are class III histone deacetylases. In mammals; the sirtuin family consists of seven proteins. In their review, S. Balaiya et al. discuss the roles of sirtuins in glaucoma, optic neuritis, and AMD, and they describe the potential of sirtuins as therapeutic targets for these diseases.

The therapeutic effects of free radical scavengers in various retinal diseases have been studied extensively. T. Masuda et al. provide a comprehensive review of the therapeutic potential of edaravone, a free radical scavenger, in glaucoma, AMD, DR, and retinal vein occlusion. Edaravone can protect against N-methyl-d-aspartate-induced retinal thinning by reducing OS and inhibiting activation of the JNK and p38 MAP kinase pathways, suggesting that edaravone may have therapeutic activity in glaucoma. Edaravone may also slow the progression of AMD, not only through its anti-OS and antiapoptotic effects but also through the suppression of VEGF-induced endothelial cell proliferation. T. Masuda et al. indicate that edaravone may slow the progression of DR by attenuating the suppression of brain-derived neurotrophic factor. In a clinical trial, edaravone following arteriovenous sheathotomy was effective against macular edema associated with a branch retinal vein occlusion and improved the best-corrected visual acuity. These findings suggest that edaravone may be useful for treating retinal diseases associated with OS.

Another important therapeutic strategy for OS-associated retinal diseases is elevation of antioxidant enzymes. Y. Nakagami reviews the therapeutic potential of small molecule compounds that can activate nuclear factor erythroid 2-related factor 2 (Nrf2). Nrf2 is a redox-sensitive transcription factor that binds to antioxidant response elements located in the promoter region of genes encoding many antioxidant enzymes and phase II detoxifying enzymes. Y. Nakagami recently developed a novel Nrf2-activating small molecule compound; here, they demonstrate its therapeutic effects on various retinal diseases associated with OS.

Finally, Y. Nishimura and H. Hara focus on recent advances in high-throughput technologies that have facilitated the collection of multilevel omics data. Integration of the knowledge gained from omics databases can be used to generate disease-related biological networks and to identify potential therapeutic targets within the networks. The authors provide an overview of integrative approaches in the drug discovery process and provide simple examples of how the approaches can be exploited to identify OS-related targets for retinal diseases.

We hope that this special issue will stimulate further efforts to understand retinal diseases at the systems level, with the hope of finding new preconditioning and therapeutic strategies to prevent or treat retinal pathologies.

